# Oxytocin-induced anxiogenic behavior in juvenile male rats

**DOI:** 10.1080/19768354.2021.1995485

**Published:** 2021-10-31

**Authors:** Minji Jang, Taesub Jung, Miseon Kang, Jeongyeon Kim, Jihyun Noh

**Affiliations:** a Department of Science Education, Dankook University, Yongin-si, Republic of Korea; b Korea Brain Research Institute (KBRI), Daegu, Republic of Korea

**Keywords:** Anxiety, emotional behavior, nicotine aversion, oxytocin, stress

## Abstract

Oxytocin (OT) is considered beneficial to mental health owing to its anxiolytic, prosocial, and anti-stress effects; however, the adverse effects of OT have been controversial, such as its potentially anxiogenic actions. Although OT influences drug abuse and reciprocally affects vulnerability to drug use, the relationship between OT’s anxiogenic working and nicotine preference intake has not been clearly defined. To clarify this issue, the effect of acute peripheral administration of OT on anxiety and nicotine preference was investigated in juvenile male rats. Anxiogenic behaviors were noticeably increased in OT-administrated rats, with an increase in serum corticosterone levels. Moreover, increased anxiety-like behaviors and corticosterone levels were observed in the OT analog carbetocin-injected rats. In the nicotine preference test, the rats’ aversive responses to initial nicotine choice and preference were not significantly different between saline-injected and OT-injected rats. However, when administered with OT, there was a significant negative correlation between anxiety-like behavior and low-dose nicotine consumption. Collectively, these results provide evidence that acute OT exposure could induce anxiogenic behavior with corticosterone augmentation, contributing to the attenuation of nicotine preference. This suggests that both aspects of OT, as well as their benefits and drawbacks, should be considered.

## Introduction

Oxytocin (OT) is a neuropeptide that evokes a range of peripheral and central effects on reproductive or sexual behavior, mood, emotions, and socialization. OT administration is generally considered beneficial for mental health owing to its anxiolytic, prosocial, and anti-stress effects (Broadbear et al. 2014), and has received increasing interest as a potential treatment for addiction (McGregor and Bowen 2012). However, OT has also shown anxiogenic effects under some conditions (MacDonald and Feifel 2014; Peters et al. 2014). OT promotes territoriality, aggression, and other defensive behaviors toward out-group members, complicating any straightforward interpretation of OTs’ socio-emotional effects. Using region-specific manipulations of the murine OT receptor gene, the lateral septum was identified as the region of the brain that mediates the fear-enhancing effects of OT (Peters et al. 2014). In humans, OT administration has been shown to facilitate fear conditioning (Eckstein et al. 2016), enhance the startle reflex towards unpredictable shocks (Grillon et al. 2013), and potentiate acoustic startle responses after exposure to negative emotional pictures (Striepens et al. 2012).

OT has been associated with a reduction in drug-induced physical and emotional withdrawal symptoms, such as self-administration, tolerance, anxiety, and depressive behavior (McGregor and Bowen 2012). However, OT is suggested to be an endocrinological agent with the potential to increase nicotine self-administration in rats in social settings (Chen et al. 2011). While OT reduces withdrawal behaviors induced by chronic nicotine exposure, it is not effective in attenuating withdrawal-induced anhedonia (Manbeck et al. 2014), suggesting that its protective role against noxious properties during initial nicotine exposure may be a risk factor for additional addictive nicotine abuse. Because nicotine has both rewarding and noxious properties in that it not only stimulates a reward system but also induces unpleasant nicotine aversive behavior (Frahm et al. 2011), nicotine aversion is also a major mechanism for nicotine addiction. In our previous study, during the nicotine consumption test, peripherally injected OT not only increased nicotine intake significantly but also alleviated nicotine aversion after acclimation to nicotine solution in a concentration-dependent manner (Lee et al. 2017). However, nicotine aversive behaviors in the OT-administered group were not determined, particularly regarding anxiety.

Despite the possibility that OT may increase anxiety or nicotine preference behavior, studies on the correlation between OT-induced increases in anxiety and nicotine-induced alterations are lacking. Therefore, in this study, we focused on the effects of OT on anxiety-like behavior and nicotine aversion by identifying changes in anxiety and nicotine preference behavior in OT-exposed male rats. To further probe the effects of OT on anxiety, we exposed naïve male rats to OT and OT receptor agonist and verified their anxiety behavior and blood corticosterone levels.

## Materials and methods

### Animals

**Figure 1.**
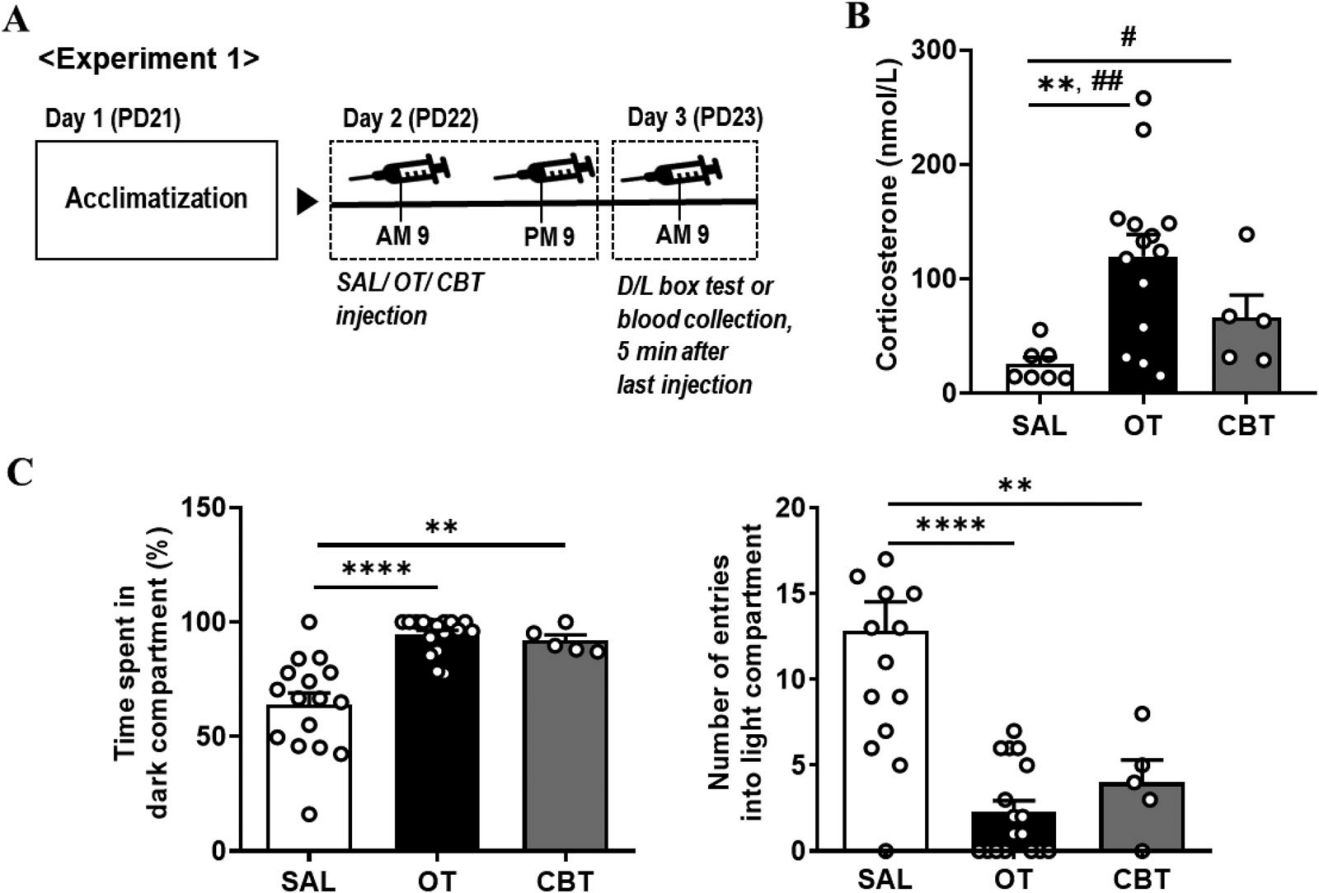
Alteration of anxiety-like behaviors and corticosterone levels in the blood by oxytocin (OT) and OT receptor agonist injection. (A) The flowchart shows the schematic timeline of experiment 1 procedures. SAL, saline; OT, oxytocin; CBT, OT receptor agonist, carbetocin; PD, postnatal day; D/L box test, dark/light box test. (B) Change of serum corticosterone levels by OT or CBT injection (One-way ANOVA, Tukey’s post-hoc test, **P < 0.01; Unpaired *t*-test, ^#^*P* < 0.05, ^##^*P* < 0.01). (C) The time spent in the dark compartment (*Left*) and the number of entries into the light compartment (*Right*) after OT or CBT injection (One-way ANOVA, Tukey’s post-hoc test, **P < 0.01, ****P < 0.0001). Data are presented as mean ± standard error mean.

**Figure 2.**
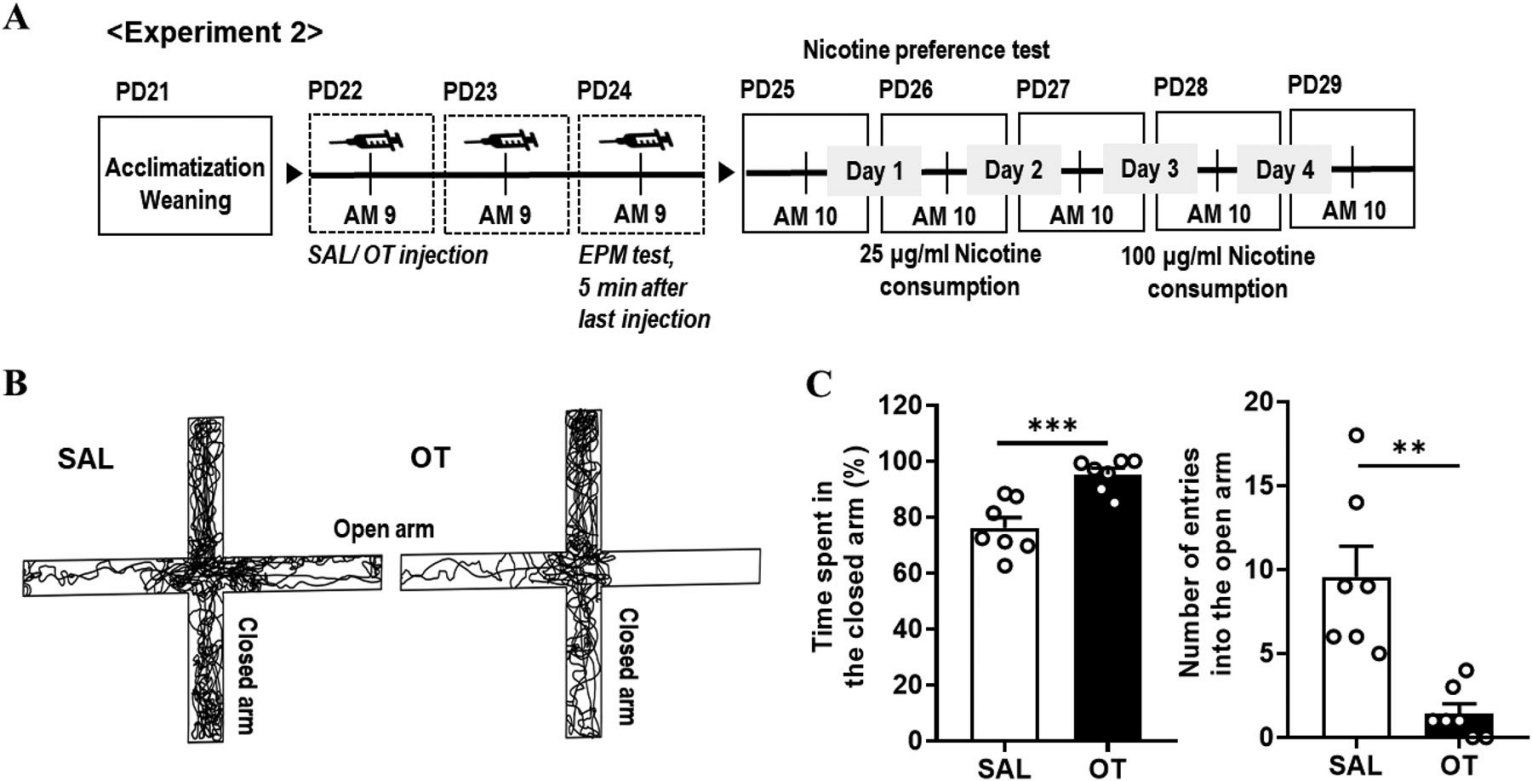
Effect of oxytocin (OT) on nicotine preference and anxiety-like behavior. (A) Schematic timeline presenting a schedule of experiment 2 (SAL, saline; OT, oxytocin; PD, postnatal day; EPM, elevated plus maze). (B) Exploration paths of representative SAL- and OT-exposed rats in the EPM test. (C) *Left*, Time spent in the closed arm. *Right*, Number of entries into the open arm (Unpaired *t*-test, **P < 0.01, ***P < 0.001).

All animal studies were conducted in accordance with the Dankook University Ethics Committee’s Guidelines for the Care and Use of Laboratory Animals (DKU-18-009; DKU-21-012). Sprague–Dawley male rats (postnatal day, PD17) obtained from Orient Bio (Seongnam, Republic of Korea) were housed in Plexiglas cages with wood bedding in an air-conditioned room at 23 ± 1°C (45 ± 5% humidity) with a standard 12-h light/dark cycle (light on 9:00–21:00). The experimental schematic timeline is shown in Figure 1(A) (experiment 1) and Figure 2(A) (experiment 2). Basically, all tests were performed between 9:00 and 12:00. The food was provided unlimitedly, and the weight was measured daily during the experiment. In experiment 1, the dark/light (D/L) box test or blood collection were examined within 5 min after last injection of saline, OT, or carbetocin (CBT). In experiment 2, the elevated plus maze (EPM) test 5 min after last saline or OT injection was performed, and then the nicotine preference test was performed 24 h after EPM test.

### Drugs

In the nicotine preference test, 25 and 100 μg/mL of nicotine was used in the drinking water. For OT (Tocris) and CBT (Tocris) administration, rats were subcutaneously injected with 1 mL/100 g of saline (0.9% NaCl), OT (1 mg/kg) (Lee et al. 2017), or CBT (6.4 mg/kg) (Zanos et al. 2014), according to their respective assigned groups, at PD 22–23, three times at 12-h intervals in experiment 1(Figure 1(A)). In experiment 2, saline (0.9% NaCl), OT (1 mg/kg) were subcutaneously treated to assigned rats three times at 24-h intervals during PD 22–24 (Figure 2(A)).

### Nicotine preference test: oral nicotine consumption by two-bottle free-choice paradigm

Rats were provided with nicotine and water in two bottles for 4 days. The starting concentration of nicotine (nicotine hydrogen tartrate salt, Sigma-Aldrich) was 25 µg/mL per bottle during the initial 2 days of nicotine exposure, which was changed to 100 µg/mL for the next 2 days. The positions of the two bottles were interchanged every 24 h to overcome any positional preference in the rats. The volume of water and nicotine intake from each bottle was calculated at 24 h between 10:00 and 12:00. Each fluid intake was calculated by dividing the amount of solution consumed during the experiment by 24 h and the weight of each individual rat.

Nicotine consumption (ml/kg/h) = amount of nicotine solution consumed (ml)/ rat body weight (kg)/ experiment time (24 h)

Nicotine preference was calculated as the ratio of the volume of nicotine solution consumed divided by the total fluid intake for each rat.

Nicotine preference (%) = [nicotine consumption (mL) / total fluid consumption (mL)] × 100.

### Dark/light box (D/L box) test

In a D/L box (40 × 20 × 31 cm), rats were placed in the dark compartment (approximately 1 Lx) through the front door (8 × 8 cm). The middle door between the dark and light compartments (approximately 100 Lx) was opened 3 s after the front door was closed. The behaviors of the rats were analyzed for 15 min and recorded using a camcorder (HMX-H304BD, Samsung, Republic of Korea). The percentage of time spent in the dark compartment and the number of entries into the light compartment were calculated for each rat to serve as a reliable measure of anxiety.

### Elevated plus maze (EPM) test

An EPM test was conducted to examine anxiety-like behaviors in rats. The assigned rats were last injected with either saline or OT, 5 min before the test. The rats were placed facing a closed arm in the EPM. Rats were recorded using a camcorder (HMX-H304BD) for 10 min, as they freely explored the maze. Behavioral analysis was performed blindly by more than two experimenters. The percentages of open arm entries (open/total entries × 100) and the time spent in the open arms (open/[open + close time] × 100) were calculated for each rat (Jung and Noh 2021).

### Corticosterone enzyme-like immunoassay (CORT-ELISA)

We checked the level of corticosterone in the serum to confirm the stress response (Park et al. 2020). On PD 23, the rats were sacrificed for blood collection after last drug injection. The samples were centrifuged at 1350 rpm for 10 min at 25°C, and blood serum was harvested. Blood serum samples were stored at −80°C before measurement. Serum corticosterone level was measured by ELISA Kit (IBL International GmbH, Flughagenstr. 52A, 22335 Humberg, Germany, RE52211) according to manufacturer’s instruction. Briefly, 20 μl of sample serums and standard solutions were added to the already pre-coated antibody plate provided with the kit and incubated for 60 min at room temperature. The reaction was terminated and followed by washing, 100 μl of TMB substrate was added and incubated for 15 min without shaking. The reaction was stopped by adding 50 μl of TMB stop solution. Absorbance was read at 450 nm by using the multi-microplate reader (Spectramax iD5 multi-mode microplate reader, Molecular Devices, USA). Sample concentrations for corticosterone were calculated from a standard curve by using GraphPad software (GraphPad prism 8.0, La Jolla, CA).

### Statistical analyses

The data were analyzed using Prism8 software (GraphPad Software, Inc., USA) and expressed as the mean ± standard error of the mean. Statistical significance was estimated using the unpaired two-tailed Student’s t-test to compare the differences between the two groups. For multiple comparisons, one-way analysis of variance (ANOVA) followed by post-hoc Tukey’s multiple comparison test, and two-way ANOVA followed by Bonferroni post-hoc test were used. Correlations between the percentage of time spent in the dark compartment and nicotine consumption were performed using Pearson’s correlation test. Asterisks indicate statistically significant differences among the groups.

## Results

### Induction of anxiety-like behavior by OT

To clarify the effect of OT on anxiety in juvenile male rats, we determined the alteration of anxiety-like behavior by OT in experiment 1. As the half-life of OT is suggested to be 3–6 min (Mens et al. 1983), the tests were conducted 5 min after OT injection. When OT or OT analog CBT were injected, significantly higher serum corticosterone levels were observed compared to those in the saline (SAL) group (Figure 1(B); one-way ANOVA, F (2, 23) = 6.61, *P* = 0.0054, SAL, 25.24 ± 6.1, *n *= 7; OT, 119.7 ± 19.11, *n *= 14; CBT, 65.96 ± 19.85, *n *= 5; **P < 0.01, Tukey’s post-hoc test; ^#^*P* < 0.05, ^##^*P* < 0.01, unpaired t-test). In a D/L box test, after both OT and CBT injection, the time spent in the dark compartment was significantly longer than that in the SAL group based on one-way ANOVA (Figure 1(C) *Left*; F (2, 35) = 19.79; *P* < 0.0001; SAL, 63.86 ± 5.16%, *n *= 16; OT, 94.51 ± 1.85%, *n *= 17; CBT, 91.98 ± 2.44%, *n *= 5; ****P < 0.0001, **P < 0.005; Tukey’s post-hoc test). Similar results were obtained with the number of entries into the light compartment; rats from the OT and CBT groups showed fewer entries than those from the SAL group, as determined by one-way ANOVA (Figure 1(C) *Right*; F (3, 35) = 13.36; *P* < 0.0001; SAL, 12.81 ± 1.7, *n *= 16; OT, 2.29 ± 0.64, *n *= 17; CBT, 4 ± 1.3, *n *= 5; ****P < 0.0001, **P < 0.005; Tukey’s post-hoc test). This suggests that an immediate response to OT may induce an increase in anxiety. As anxiety and serum corticosterone level are closely related, corticosterone levels may be the basis for this effect. In conclusion, OT administration within a short-term interval until the test significantly increased anxiety and corticosterone levels in rat blood.

### Correlation between nicotine preference and anxiety by OT

In experiment 2, the correlation between nicotine preference behavior and anxiety-like behavior in OT-administered rats was demonstrated (Figure 2(A)). Similar to experiment 1, anxiety-like behavior increased significantly in OT-exposed rats in the EPM test (Figure 2(B,C). In the time spent in the closed arm analysis, there was a significant increase in the OT-exposed group compared to the SAL group (Figure 2(C) *Left*; SAL, 76.15 ± 3.67, *n *= 7; OT, 95.31 ± 2.16, *n *= 7; ***P = 0.0007, unpaired t-test). The OT-exposed group showed a significant decrease in the number of entries into the open arm compared to that in the SAL group (Figure 2(C) *Right*; SAL, 9.57 ± 1.81, *n *= 7; OT, 1.43 ± 0.57, *n *= 7; **P = 0.0011, unpaired t-test).

**Figure 3.**
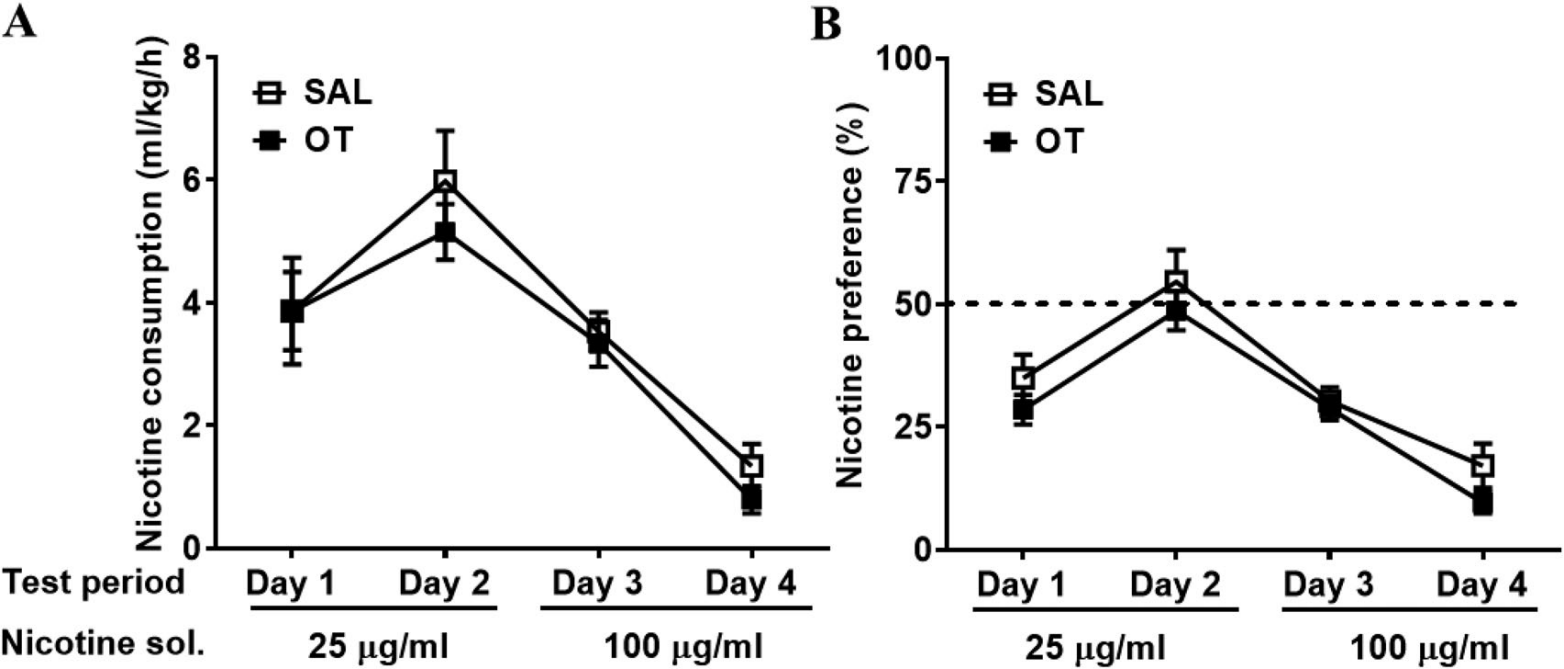
Nicotine consumption (*A*) and nicotine preference (*B*) in the two-bottle test between SAL and OT-exposed rats (Two-way ANOVA).

We verified the nicotine preference for low (25 μg/mL) and high (100 μg/mL) nicotine solutions in SAL- and OT-injected rats, using the two-bottle free-choice paradigm. In nicotine consumption analysis, the effects of group (F (1, 12) = 0.62, *P* = 0.45) and day × group interaction (F (3, 36) = 0.28, *P* = 0.84) were not significant; however, the day (F (3, 36) = 27.95, *P* < 0.0001) was significant among groups, as determined using two-way ANOVA (Figure 3(A)). In nicotine preference analysis, the effects of group (F (1, 12) = 2.36, *P *= 0.15) and day × group interaction (F (3, 36) = 0.27, *P* = 0.85) were not significant; however, the day (F (3, 36) = 36.70, *P* < 0.0001) was significant among groups, as determined using two-way ANOVA (Figure 3(B)). Collectively, there was no significant difference in nicotine consumption and preference between SAL- and OT-exposed rats.

**Figure 4.**
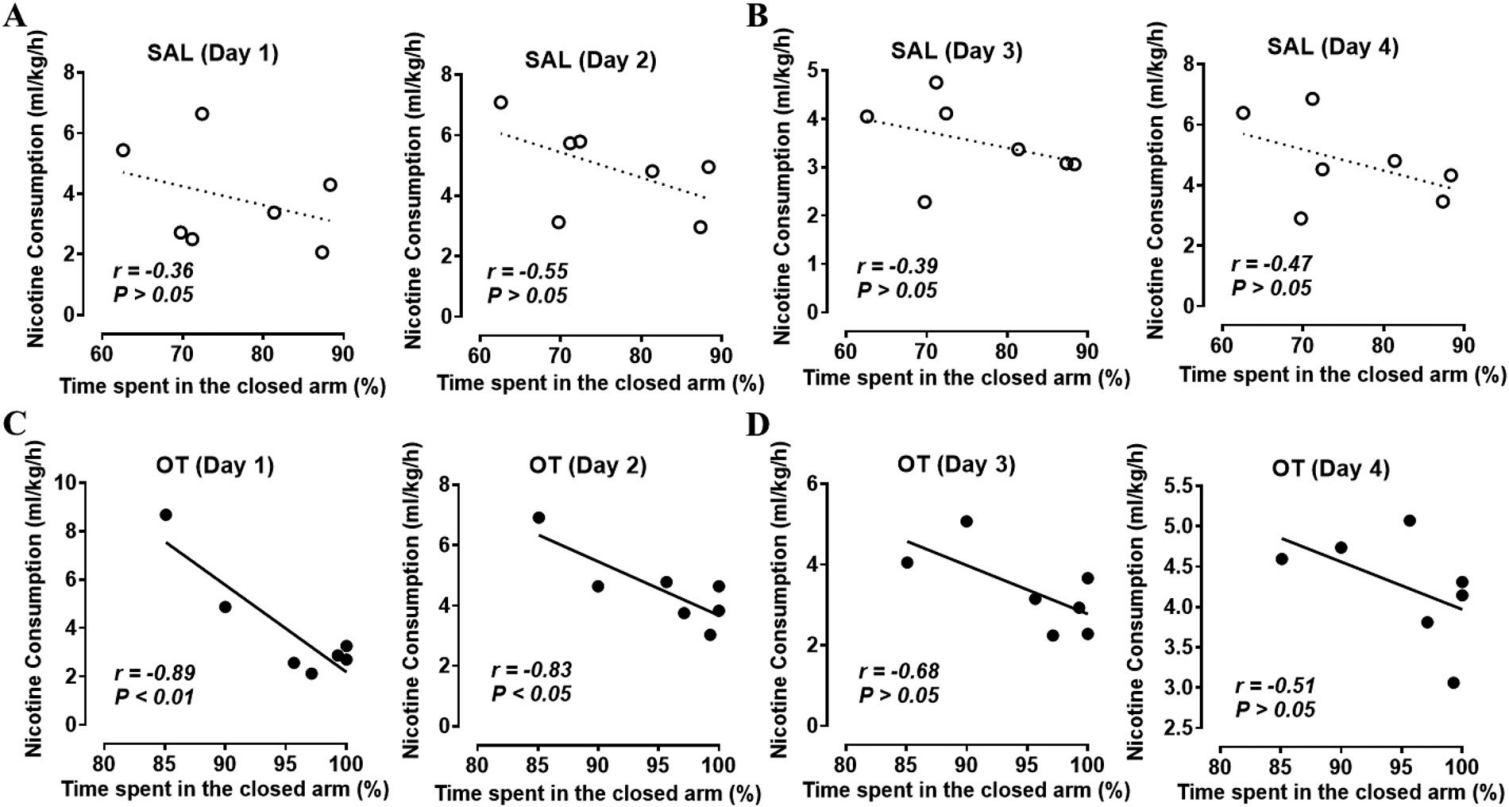
Correlation analysis between nicotine consumption and time spent in the closed arm. (A, C) 25 μg/ml nicotine consumption test. (B, D) 100 μg/ml nicotine consumption test. (A, B) SAL-exposed rats. (C, D) OT-exposed rats. SAL, saline; OT, oxytocin

To elucidate the role of OT in the correlation between nicotine intake and anxiety, we compared the correlation between nicotine consumption and the time spent in the closed arm using EPM (Figure 4). In SAL-exposed rats, there were significant correlations in neither the 25 μg/mL (Figure 4(A); *Left*, Day 1, *r* = −0.36, *P* > 0.05, *n* = 7; *Right*, Day 2, *r* = −0.55, *P* > 0.05, *n* = 7) nor the 100 μg/mL nicotine consumption test (Figure 4(B); *Left*, Day 3, *r* = −0.39, *P* > 0.05, *n* = 7; *Right*, Day 4, *r* = −0.47, *P* > 0.05, *n* = 7). In OT-exposed rats, the 100 μg/mL nicotine consumption test (Figure 4(D); *Left*, Day 3, *r* = −0.68, *P* > 0.05, *n* = 7; *Right*, Day 4, *r *= −0.51, *P* > 0.05, *n* = 7) showed no correlation, while the 25 μg/mL nicotine consumption test showed a significant correlation (Figure 4(C); *Left*, Day 1, *r* = −0.89, *P* < 0.01, *n* = 7; *Right*, Day 2, *r *= −0.83, *P *< 0.05, *n* = 7). Altogether, when administered with OT, there was a significant negative correlation between anxiety-like behavior and low-dose nicotine consumption.

## Discussion

This study suggests that OT can increase anxiety-like behavior and contribute to a significant correlation between anxiogenic behavior and low-dose nicotine intake. Increased anxiety was found to be an immediate response following OT exposure, and the blood corticosterone level was significantly higher than that in the control group (Figure 1). A study suggested that OT administration suppresses stress and thus decreases corticosterone levels (Heinrichs et al. 2003), while the other suggests that corticosterone administration increases plasma OT levels (Kalin et al. 1985), as beneficial effects of OT administration on stress coping and emotional memory. However, it was shown that ACTH and corticosterone levels increased 30 min after 1 mg/kg subcutaneous OT injection for 5 days (Petersson et al. 1999). Our results also show an increase in corticosterone levels following OT or OT receptor agonist administration, and this phenomenon may suggest inducing an increase in transient anxiety caused by OT. It is also suggested that OT can create transient tense situations of stress, such as a fight or flight as anxiety is associated with stress responses.

Studies in which the effects of OT on emotional behavior, such as anxiety or fear are contradictory depending on the development, dosage, and injection time have been presented in previous studies. Developmentally, TGOT which is selective OT receptor agonist in the basolateral amygdala after the retrieval of fear conditioning impairs fear extinction at juvenile (P27) male rats, but not in adult (P60) (Kritman et al. 2017). Depending on the OT doses, chronic intracerebroventricular (i.c.v.) infusion with OT at high (10 ng/h) dose leads to increased anxiety, but not at low (1 ng/h) dose (Peters et al. 2014). In even intraperitoneal (i.p.) OT injected male C57BL/6J mice (6 weeks), there was no change in anxiety at low concentration (0.1 mg/kg), but anxiety increased at high dose (1 mg/kg)(Sakamoto et al. 2019). It is consistent with our anxiety results using the 1 mg/kg oxytocin for juvenile rat (Figure 1(C)). Interestingly, social interaction duration in social interaction test increased when OT of the same concentration (1 mg/kg) was injected 24 h ago, but that duration decreased when injected 10 min before the experiment (Sobota et al. 2015; Sakamoto et al. 2019). In our previous paper, it was confirmed that OT reduced aversive toward nicotine and anxiety (Lee et al. 2017). There is no difference in the age of the rats, but there is a difference in that the anxiety confirmation time is within 5 min after OT injection in this experiment, whereas in the previous experiment, it is within 12 h after OT injection. This experiment measured nicotine preference after OT administration, but the previous experiment has a difference in measuring nicotine preference simultaneously during OT administration. However, in both cases, there was no difference in nicotine preference at the same OT concentration.

In this experiment, OT showed a significant negative correlation between anxiety-like behavior and low nicotine intake. This can be explained by a decrease in the intake of low-concentration nicotine, that is, an increase in aversion to nicotine, as OT-induced anxiety increases (Figure 3). Consistent with our data, intranasal administration of OT increases the recollection of aversive events (Bartz et al. 2010) and startle responses to stressful stimuli (Grillon et al. 2013). Additionally, OT reactivity is associated with increased post-conflict anxiety (Tabak et al. 2011). The negative risk-valence context, in which this OT risk-averse effect emerged, might have promoted anxiogenic-like effects (Patel et al. 2015). Certainly, OT has also been associated with defensive responses in potentially dangerous situations when facing ‘out-group’ antagonists (De Dreu et al. 2010). In line with this notion, OT has been found to reduce cooperativeness with opponents in conditions of social unfamiliarity (Declerck et al. 2010). Familiarity may be a key factor modulating the effects of OT on behavior, with OT being more anxiogenic in unfamiliar contexts and more anxiolytic in familiar contexts. These findings are consistent with the exacerbation of defensive behaviors induced by OT during social stress. This suggests that, rather than displaying a unidirectional influence on anxiety, the OT system has a modulatory role, possibly by changing the salience or emotional valence of social and nonsocial contexts.

High concentrations of OT showed an influence similar to that of vasopressin (AVP) in memory processes related to vasopressinergic receptors, suggesting that high concentrations of OT may affect AVP receptors (VR). As we could not exclude the possibility that OT binds to V_1a_Rs, we tested the rat’s anxiety behavior and measured corticosterone level, using an OT analog CBT, which has a potent and selective OTR/V_1a_R ratio of 500 − 800 (Passoni et al. 2016). We found that CBT also significantly increased anxiety behavior and corticosterone levels (Figure 1(B,C)), suggesting that the OT-induced anxiogenic behavior may be attributed to the OT system rather than the AVP system.

Sex differences in the OT system are not always uniform, often showing higher OT expression in females than in males, but higher OTR expression in males than in females (Dumais and Veenema 2016). The utilization of the central OT system has been identified as a promising strategy in translational neuroscience for the development of targeted pharmacological interventions to improve outcomes in several conditions currently lacking efficacious treatment, such as autism spectrum disorder or Prader-Willi syndrome. Furthermore, as OT is recognized as having endocrine and paracrine roles in male reproduction, exogenous administration of OT and OT antagonists may also have a role in treating pathologies related to male reproduction, specifically improving sperm parameters and in the treatment of prostate disorders (Thackare et al. 2006). In the future, since peripheral OT injections have significant therapeutic potential to improve total psychopathology or alleviate symptoms in some disorders, OT application will be expanded. It is also necessary to investigate the mechanism by tracking changes in specific brain regions at the molecular level by oxytocin through transcriptome analyses (Lee et al. 2019).
